# Preparation and Properties of SiBCO Aerogel and Its Composites

**DOI:** 10.3390/nano9010040

**Published:** 2018-12-29

**Authors:** Xiafei Li, Junzong Feng, Jie Yin, Yonggang Jiang, Jian Feng

**Affiliations:** 1 Science and Technology on Advanced Ceramic Fibers and Composites Laboratory, College of Aerospace Science and Engineering, National University of Defense Technology, 109 De Ya Rd, Changsha 410073, China; yayasummer0529@163.com (X.L.); zhangzhen12a@126.com (Y.J.); 2 Jiuquan Satellite Launch Centre, 27 Branch Post Office, Jiuquan 732750, China; yinjie365044@163.com

**Keywords:** SiBCO aerogel, thermal insulation, mechanical property, high-temperature resistance

## Abstract

To obtain new high-temperature resistant composites that can meet the requirements of aircraft development for thermal insulation and mechanical properties, SiBCO aerogel composites were prepared by sol-gel, supercritical drying and high-temperature pyrolysis with trimethyl borate (TMB) or phenylboronic acid (PBA) as the boron source and mullite fiber as reinforcement. The structure and composition of the SiBCO aerogel and its composites were characterized with SEM, FT-IR, ICP and nitrogen adsorption tests. The specific surface area of the SiBCO aerogel is 293.22 m^2^/g, and the pore size is concentrated in the range of 10–150 nm. The mechanical properties, the thermal insulation properties and the temperature resistance were also studied. Due to the introduction of boron, the temperature resistance of SiBCO aerogel composites is improved greatly, and the service temperature of composites reached 1773 K. When n (TMB)/n (TEOS) = 1/1, the temperature resistance of the composites is the best. After heating in air at 1773 K for 30 min, the shrinkage of SiBCO aerogel composites is only 2.45%, and the thermal conductivity of the composites is 0.138 W/(m·K) at 1773 K. In addition, the type and amount of catalyst also have certain effects on the mechanical properties and temperature resistance of the composites.

## 1. Introduction

Aerogel is considered to be the solid material with the lowest thermal conductivity [1,2,3], among which SiO_2_ aerogel is studied most comprehensively and has been applied in military, aeronautic and civil uses due to its thermal conductivity that can be as low as 0.013 W/(m·K) [4,5,6,7]. However, the effect of sintering is obvious when the usage temperature is over 1073 K, which leads to a serious degradation of the heat insulation property of the material [8,9,10].

Referring to research on SiCO ceramics [11,12,13,14], researchers believe that if amorphous Si-O structures in SiO_2_ aerogels are partly replaced by Si-C structures to form Si-C-O structures, the SiCO aerogels obtained will not only show lower thermal conductivities than those of SiCO ceramics but also show better thermal stability and mechanical properties [15,16,17]. Manuel W et al. [18] synthesized 1,3,5-dimethoxymethyl cyclohexanes as precursors with chloromethyltrimethoxysilane, and a polymer was prepared with ethylene glycol replacing methanol. SiCO aerogel was obtained after phase separation, CO_2_ supercritical drying, and pyrolysis at 1273 K under argon atmosphere. However, the low density and high porosity of the SiCO aerogel made it brittle. Zhao et al. [19] synthesized the SiCO precursor sol with tetraethyl orthosilicate (TEOS) as the silicon source and dimethyldiethoxylsilane (DMDES) as the carbon source and further prepared SiCO aerogel composites with reinforced fibers through sol-gel, supercritical drying and high-temperature pyrolysis. The composites present excellent thermal insulation properties and high-temperature resistance.

To meet the increasing requirements for heat resistance performance, the augmentation of the highest utility temperature of aerogels has been the main topic in recent years. Relevant studies show that SiBCO ceramics and SiBCO high-temperature resistant glass prepared by the introduction of boron in the SiCO material system show better high-temperature thermal stability [20,21,22,23]. Soraru et al. [24] discovered for the first time in 1997 that during the process of ceramics preparation by the precursor transition method, the addition of boron in SiCO gels could prominently improve the ceramic productivity, and in the follow-up studies, researchers prepared varieties of SiBCO materials by changing raw materials and the experimental procedure. The studies carried out by Muralidharan et al. [25] indicated that the crystallization of material has a tendency to decrease after heating at 1773 K, as the content of trimethyl borate (TMB) increases in the porous ceramic preparation process. Tamayo et al. [26] prepared the SiBCO glass fiber using MTES, DMDES and boronic acid as raw materials and found that the addition of boron element can enhance the thermal stability of materials.

Until now, studies of SiBCO materials mostly focused on porous ceramics and high-temperature resistant glass, and there is no relevant literature about SiBCO aerogels. According to the preparation methods of SiBCO materials reported in papers combined with the previous studies of SiCO aerogel thermal insulation composites by our research group [27,28], SiBCO aerogels and their composites reinforced by mullite fiber are prepared in this paper using TEOS, DMDES and trimethyl borate (TMB) or phenylboronic acid (PBA) as raw materials. The introduction of boron can greatly improve the temperature resistance of SiCO aerogel composites due to the formation of a SiBCO glassy antioxidant film on the surface of the composites at high temperature. The effects of raw materials and their amount on the microstructure, composition and properties of the SiBCO aerogel composites were investigated.

## 2. Experiment

### 2.1. Materials

Tetraethoxysilane (TEOS) was obtained from Shanghai Wulian Chemical Factory Co., Ltd., China and Methyldimethoxysilane (DMDES) was purchased from Wuhan Yi Hua Cheng Technology Development Co., Ltd., China. Trimethyl borate (TMB) or phenylboronic acid (PBA) were obtained from Sinopharm Chemical Reagent Co., Ltd., Beijing, China. Nitric acid (HNO_3_), ammonia (NH_4_OH), ethylenediamine (EN) and ethanol (EtOH) were all bought from the Beijing Institute of Chemical Reagents. The mullite fibers were purchased from Hunan Jiuhua Carbon High-Tech Co., Ltd., China, and used as the reinforcement. HNO_3_ and NH_4_OH (or EN) were diluted to 0.1 mol/L and 1 mol/L, respectively, and the other chemical reagents were used as received without purification.

### 2.2. Preparation

The SiBCO precursor sol was prepared through the method of acid-base two-step catalysis with TEOS as the silica source, DMDES as the carbon source, and TMB or PBA as the boron source. TEOS and DMDES were mixed with a certain amount of H_2_O and EtOH and stirred evenly. Then, HNO_3_ was added to the mixed solution as an acid catalyst to sufficiently hydrolyze TEOS and DMDES. Next, TMB or PBA was added and further mixed. After that, NH_3_•H_2_O or EN was slowly dropped into the solution as the basic catalyst to make the hydrolysate of TEOS, DMDES and TMB (or PBA) polymerized. Then, the synthesized SiBCO sol precursor or mullite fibers/SiBCO sol was hermetically placed in an oven with a constant temperature at 338 K to gel and age for 1 day. To prevent gel cracking caused by solvent evaporation, EtOH should be added on the gel surface in a timely manner during ageing. SiBCO aerogel and its composites were prepared by supercritical drying and pyrolysis at 1473 K. The molar ratio of TEOS/DMDES/TMB(PBA)/H_2_O/EtOH/HNO_3_/NH_3_·H_2_O(EN) = 1/0.5~3/0~1/16.7/4.6~16/0.1/0~0.1 was selected.

### 2.3. Characterization

Fourier transform infrared spectroscopy (FT-IR) and inductively coupled high-frequency plasma spectrometry (ICP) were used to analyze the elementary composition and distribution of SiBCO aerogel on the micro-scale. The aerogel pore structure was analyzed through nitrogen adsorption testing, and the bending strength and compressive strength of the composites were determined by a mechanical testing machine. The microstructures of the composites were observed by a Hitachi S4800 field emission scanning electron microscope (FE-SEM) after coating the samples with a thin gold layer. The thermal conductivities at room temperature of SiBCO aerogel composites were determined based on a hot-disk method, and the thermal conductivities at high temperature were measured by a plane table thermo-conductivity meter. A temperature resistance test was carried out in a KBF700 furnace in an oxidizing environment at different temperatures for 30 min. The linear shrinkage of the composites was calculated from the dimensions before and after tests.

## 3. Results and Discussion

The SiBCO aerogel and its composites were synthesized through the sol-gel method, while the hydrolysis and the polycondensation can be carried out simultaneously during the preparation process. Therefore, the relative rates of hydrolysis and polycondensation of TEOS, DMDES, and TMB or PBA determine the numbers and diameter of SiBCO sol particles in the final sol system and ultimately determine the mechanical properties, thermal insulation and high-temperature resistance of the materials. Through two-step acid-base catalysis [29], the hydrolysis and polycondensation reactions happen under acidic and near-neutral conditions, respectively, which can effectively control the microstructure of the aerogel and prepare a SiBCO aerogel and its heat insulation composites that meet property requirements.

**Figure 1:**
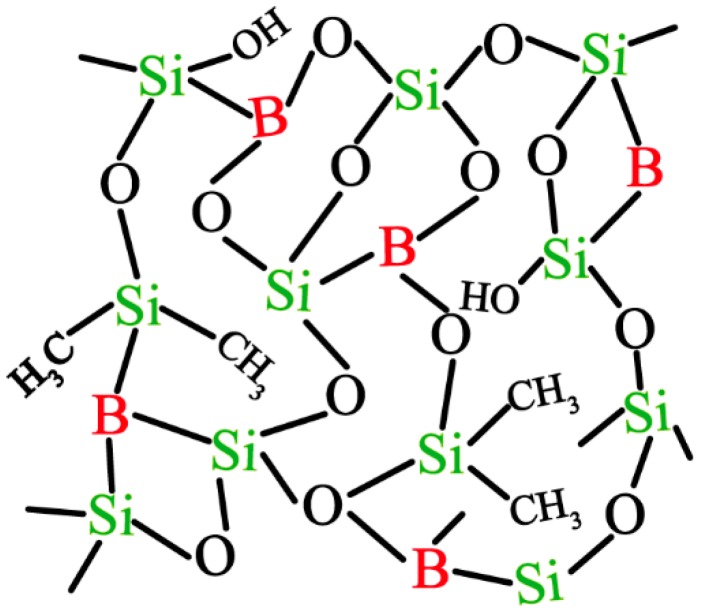
The three-dimensional network structure of SiBCO.

The hydrolysis of TEOS, DMDES and TMB to form hydroxyl under acidic conditions is required before the subsequent condensation reaction is carried out. When PBA is the boron source, it can directly undergo self-polycondensation to dimer, and at the same time, it reacts with the hydrolysates of TEOS and DMDES to form Si-O-Si, B-O-B and Si-O-B bonds [30]. The dimers are further condensed and cross-linked to form a three-dimensional network structure, as shown in Figure 1.

**Figure 2:**
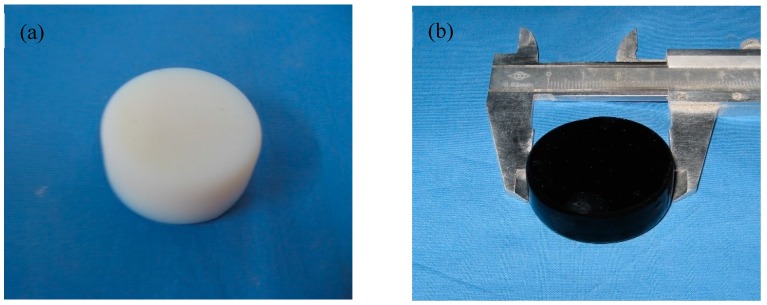
Photographs of sample before (**a**) and after (**b**) pyrolysis.

Through the pyrolysis process, the –CH_3_ is rearranged to form an amorphous structure [31]. As shown in Figure 2, the sample changes from white to black after pyrolysis and keeps a good shape without cracking.

### 3.1. Composition and Structure of SiBCO Aerogel

**Figure 3:**
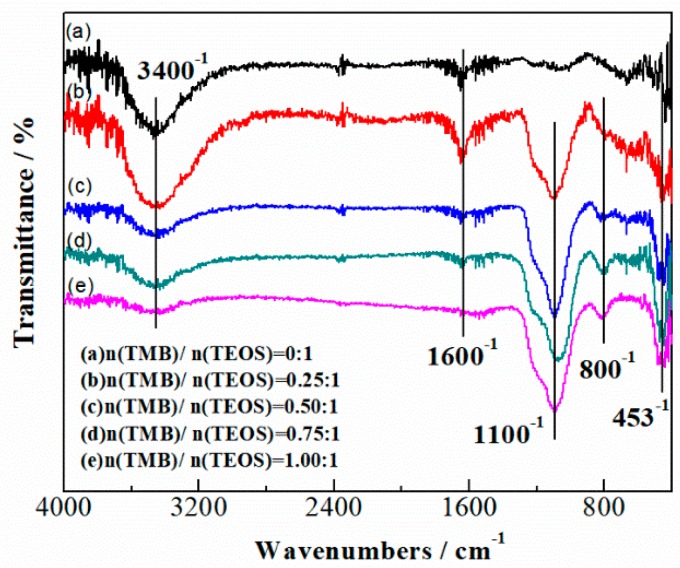
FTIR spectra of SiBCO aerogel with varying boron content.

Figure 3 shows the FTIR spectra of SiBCO aerogel with various dosages of TMB. The stretching vibration peak of the intermolecular hydrogen bond O-H near 3400 cm^−1^ weakens as the TMB content increases, while the stretching vibration peak of Si-O-B near 1100 cm^−1^ becomes stronger. The absorption peak near 1600 cm^−1^ is the stretching vibration and bending vibration peak of -OH on the aerogel surface. The absorption peak near 800 cm^−1^ and 453 cm^−1^ is caused by Si-O-Si and Si-C bonds. The above two peaks do not show any evident changes with different boron content. From the FTIR spectra, it can be considered qualitatively that an increased dosage of TMB leads to an increase of boron element content in the system, and the content of the rest of the elements do not change evidently.

**Figure 4:**
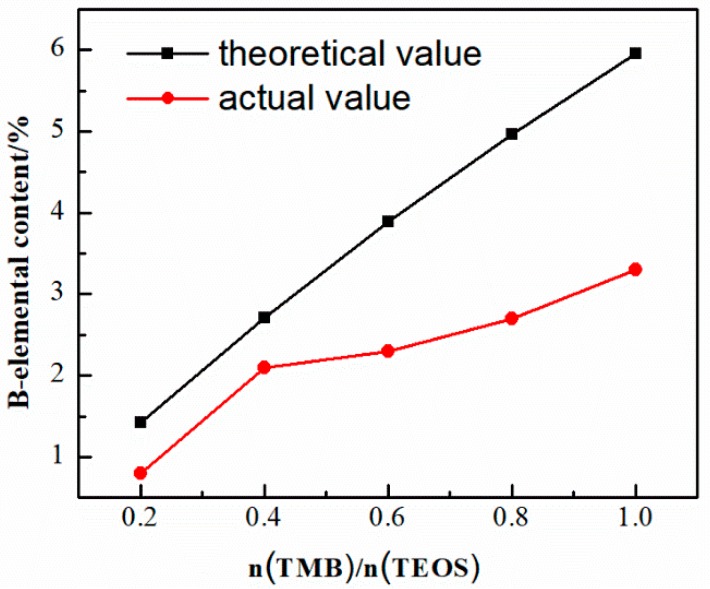
Boron element content of SiBCO aerogel with various TBM dosages.

The boron element has a small atomic number, and the content of boron in SiBCO aerogel was measured by ICP. Figure 4 indicates the curves of the theoretically calculated value and actual measured value of boron content in SiBCO aerogel varying with the amount of TMB. It can be seen that with the increase of the TMB dosage, the content of boron in the aerogel increases gradually, but the loss rate of boron increases gradually.

During the polycondensation reaction, self-polycondensation occurs between the hydrolysates of the boron source, and the products with cyclic structure will flow out with the solvent during the subsequent drying process, which is the main reason for the boron element loss. To reduce the loss of boron, the order of adding the boron source and other reactants was adjusted, and the addition rate of the boron source was controlled also.

Table 1 shows the boron element content of SiBCO aerogel prepared with various dosages of NH_3_•H_2_O or EN as the basic catalyst. It can be seen that the boron content in SiBCO aerogel prepared with EN as a catalyst is higher than that prepared with NH_3_•H_2_O as a catalyst at the same dosage. Without consideration of experiment stability and complexity of the subsequent process, the catalytic effect of EN is better than that of NH_3_•H_2_O.

**Figure 5:**
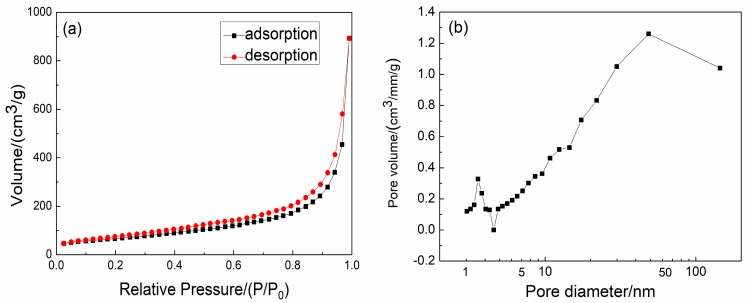
Nitrogen adsorption-desorption isotherm (**a**) and BJH pore size distribution (**b**) of SiBCO aerogel.

The nitrogen adsorption-desorption isotherm of as-synthesized SiBCO aerogel is shown in Figure 5a, which exhibits a typical type IV isotherm, indicating the presence of mesopores and macropores in the SiBCO aerogel. The adsorption volume of SiBCO aerogel in the microporous region remains basically unchanged, and the adsorption isotherm rises slowly at low relative pressure (P/P_0_). The curve has a very steep increase when P/P_0_ reaches 0.9, and the corresponding pore size is the most concentrated distribution of the aerogel, which can also be seen from the BJH pore size distribution of SiBCO aerogels in Figure 5b. Based on the calculation, the specific surface area of SiBCO aerogel is 293.22 m^2^/g; the pore size is largely focused in the range of 10–150 nm; and the most likely pore size is 48.6 nm.

**Figure 6:**
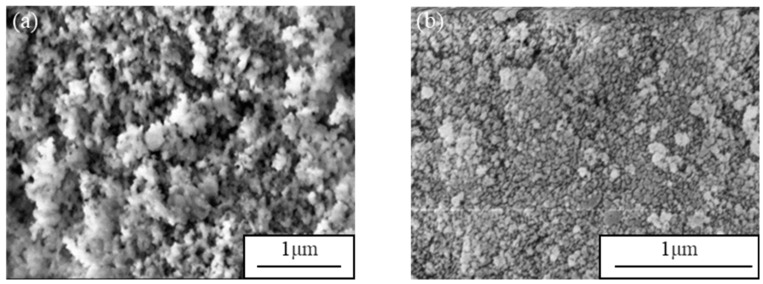
The influence of NH_3_•H_2_O on the microstructure of SiBCO aerogel. (**a**) n(NH_4_OH)/n(TEOS) = 0.02; (**b**) n(NH_4_OH)/n(TEOS) = 0.1.

To visualize the microstructure of the SiBCO aerogel we employed SEM, and the SEM images of composites with different contents of NH_3_•H_2_O are shown in Figure 6. When the amount of NH_3_•H_2_O is small (Figure 6a), the gel speed is relatively slow, and small particles formed at the beginning of gel tend to bond evenly, forming a more uniform porous structure. When the catalyst content increases (Figure 6b), the small particles rapidly condense to form larger particles during the gel, resulting in a poor porous structure for the aerogel [32].

The primary particle composition of SiBCO aerogels is a hierarchical structure [33]; i.e., the SiBCO primary particles with nanometer diameter get together to form the cluster as a secondary particle framework of SiBCO. Pores of type I (micropore) exist among primary particles, and pores of type II and III form during the arrangement of secondary particles. The volume of type II pores is insufficient to accommodate a single SiBCO secondary particle, while the SiBCO secondary particles can be accommodated in the pores of type III. The secondary particles are interconnected by polycondensation and finally form a nanoporous material with a three-dimensional structure.

### 3.2. Mechanical and Thermal Insulation Properties of SiBCO Aerogel Composites

Based on its high porosity, SiBCO aerogel is prone to brittle fracture when external forces are applied. The material absorbs energy only by the new interface formed by crack growth, so it cannot meet the requirements of processing and application. When mullite fibers are used as reinforcing phases, new energy absorption modes, such as interfacial dissociation, fiber bridging, crack deflection, broken fibers and fiber extraction, occur when the composites are loaded, which can greatly enhance the mechanical properties of composites.

**Figure 7:**
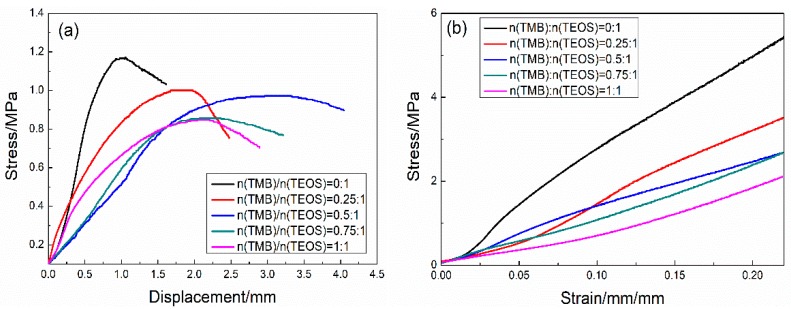
Bending stress-displacement curves (**a**) and compression stress-strain curves (**b**) of SiBCO aerogel composites with varying TMB content.

Figure 7a shows bending stress-displacement curves of SiBCO aerogel composites with varying TMB content. It can be seen that the curves show relatively evident linear characteristics in the initial stage when the loading phase is mainly an aerogel matrix, and the composites have elastic deformation. As displacement increases, the matrix continues to produce a new crack interface while fibers are stretched. The combined action of the two makes the stress gradually increase. When the load-bearing fibers are extracted or broken, the stress reaches the maximum value and then decreases rapidly. With the increase of TMB dosage, the crosslinking degree of the aerogel matrix decreases gradually, resulting in a decrease in the bending strength of the composites. The bending strength of the composites with n (TMB):n (TEOS) = 0:1 is slightly lower than that of the composites with n (TMB):n (TEOS) = 0.5:1, and the maximum bending strength is approximately 0.846 MPa when n (TMB):n (TEOS) = 1:1.

The compression stress-strain curves of SiBCO aerogel composites with varying TMB content are shown in Figure 7b, and the compressive strength gradually decreases as the TMB content increases. The compressive strength of composites with n (TMB): n(TEOS) = 1:1 is approximately 0.703 MPa when the strain is 10%, and it is 1.849 MPa when the strain is 20%, which is much lower than that with varying TMB content of composites with n (TMB): n (TEOS) = 0:1. The reason is that increasing the TMB content reduces the condensation polymerization of the aerogel matrix during the gel process and decreases the connection among particles. In addition, the loss of the boron element causes the porosity of the material to increase, so the compressive strength decreases. The SiBCO aerogel matrix has been destroyed under a small strain (<8%). When the strain continues to increase, the stress of the composites is continuously increased due to the loading of the dispersed fiber.

**Figure 8:**
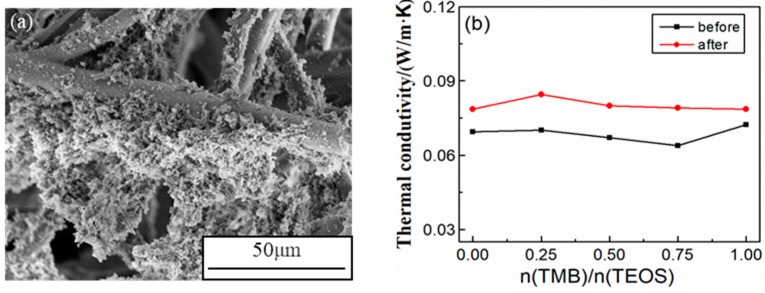
SEM image of the SiBCO aerogel composite (**a**) and its thermal conductivity (**b**) at room temperature before and after high-temperature treatment.

During the preparation of composites through the sol-gel method, the fibers are uniformly distributed due to the infiltration of sol, and the thermal bridge effect is weakened, thus reducing the solid thermal conduction of composites [34]. From the SEM image of SiBCO aerogel composites in Figure 8a, it can be seen that the aerogel matrix filled the large gaps among fibers, which evidently decreases the gaseous thermal conduction of composites [35]. Additionally, the crystal whiskers produced inside the composites during pyrolysis can effectively reduce the convective thermal transfer of the gas. Moreover, the infrared shielding property of the mullite fiber makes the radiative thermal conduction of the composites relatively low. Therefore, SiBCO aerogel composites have excellent thermal insulation properties.

The influence of TMB content on the thermal insulation property of SiBCO aerogel composites was studied, and the thermal conductivity at room temperature of the composites before and after high-temperature treatment is shown in Figure 8b. The increase of TMB content, on the one hand, reduces the SiBCO composite density, and on the other hand, it increases the porosity and pore size of the composites, resulting in the decrease of solid thermal conductivity and the increase of gaseous thermal conductivity. Based on the combination of the two factors above, the thermal conductivity of SiBCO aerogel composites decreased first and then increased, and the total thermal conductivity changed little with the increase of TMB content. After heating in air at 1773 K for 30 min, the thermal conductivity of the composites increased to some extent, but the extent of the increase gradually decreased with the increase of TMB content.

The high-temperature thermal conductivity of SiBCO aerogel composites under vacuum is shown in Table 2. It indicates that when the temperature rose from 1300 °C to 1500 °C, the thermal conductivity of the composites did not change significantly and that the thermal insulation property of SiBCO aerogel composites was stable at high temperature.

### 3.3. Temperature Resistance Performance of SiBCO Aerogel Composites

**Figure 9:**
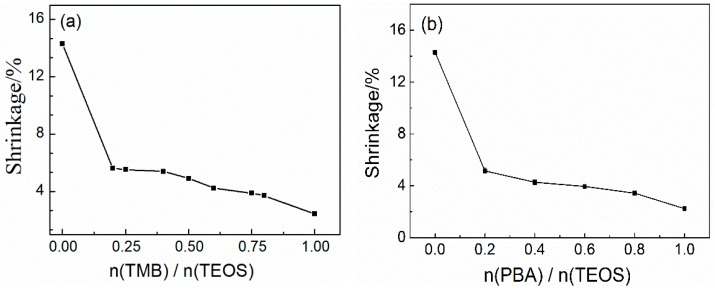
The shrinkage of the SiBCO aerogel composites with varying contents of TMB (**a**) and PBA (**b**) after heating in air at 1773 K for 30 min.

The temperature resistance of SiBCO aerogel composites prepared by different boron sources was studied. Figure 9 shows the shrinkage of the SiBCO aerogel composites with varying contents of TMB and PBA after heating in air at 1773 K for 30 min. It indicates that the introduction of boron element can significantly improve the high-temperature resistance of the composites, and with the increase of TMB or PBA content, the temperature resistance of the SiBCO aerogel composites is constantly improved. From Figure 9a, the thickness shrinkage of composites is 14.29% when n (TMB)/n(TEOS) = 0, while the shrinkage is reduced to 2.45% when n (TMB)/N (TEOS) = 1. Compared with Figure 9b and Figure 9a, it can be seen that the shrinkage of the SiBCO aerogel composites prepared with PBA as the boron source is smaller when the content of the two boron sources is the same. (When n (TMB)/n(TEOS) = 0.4, the shrinkage of the composites with PBA as the boron source is 4.27%, while it is 5.41% with TMB as the boron source. When n (TMB)/n(TEOS) = 0.8, the shrinkage of the composites is 3.43% with PBA as the boron source and 3.72% with TMB as the boron source.)

The introduction of boron resulted in the formation of a SiBCO glassy antioxidant film on the surface of the composites at high temperature, which can prevent continued oxidation of the interior of the composites. PBA contains a certain amount of carbon, which is more conducive to the formation of an antioxidant film. Subsequently, the carbon inside the composites will form graphite crystallites, which further prevents SiBCO aerogels from transforming from amorphous to a SiO_2_ structure. Therefore, the as-synthesized SiBCO aerogel composites with PBA as the boron source have better temperature resistance.

**Figure 10:**
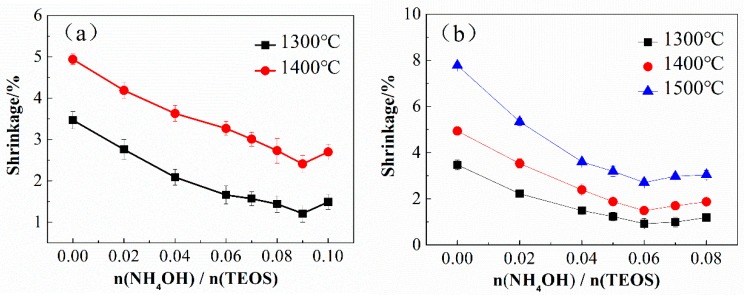
The shrinkage of the SiBCO aerogel composites with varying contents of NH_3_•H_2_O (**a**) and EN (**b**) after heating in air at different temperatures.

When using different alkaline catalysts, the shrinkage of the SiBCO aerogel composites after heating in air at different temperatures is shown in Figure 10. It can be seen from Figure 10a that the shrinkage of the composites with NH_3_•H_2_O as the catalyst reaches the minimum when n (NH_3_•H_2_O)/n(TEOS) = 0.09 after heating in air at 1300 °C or 1400 °C. As the aerogel matrix is a pearl-chain-like nanoparticle structure, an increase in the amount of NH_3_•H_2_O can enlarge the particle size. Accordingly, the shrinkage of the composites due to the sintering effect also decreases during heating at high temperature. On the other hand, when the pH value is low, TMB after hydrolysis easily reacts with NH_3_•H_2_O to form a precipitate with poor solubility. The decrease of boron content in the material system will weaken the temperature resistance of the SiBCO aerogel composites. When n (NH_3_•H_2_O)/n(TEOS) > 0.09, the particle size continues to increase, but the temperature resistance of the composites decreases sharply because of the excessive loss of boron element. Based on both factors, the shrinkage of the SiBCO aerogel composites increases slightly.

As shown in Figure 10b, when using EN as a catalyst, the shrinkage of the SiBCO aerogel composites after heating in air at different temperatures is the smallest when n (EN)/n(TEOS) = 0.06. Similar to the effect of NH_3_•H_2_O as a catalyst, at this moment, the increase of particle size and the loss of boron element have the best effects on the temperature resistance of the SiBCO aerogel composites.

In addition, it can be found from Figure 10 that the SiBCO aerogel composites prepared with EN as a catalyst have better high-temperature resistance when the dosages of the two catalysts are the same. The reason is that the reaction products produced by the hydrolysis of TMB and NH_3_•H_2_O are easy to precipitate, which causes serious boron element loss during the preparation process, so the finally synthesized SiBCO aerogel has a lower content of boron element. When EN is used as a catalyst, the boron element remains relatively stable because most TMB is condensed in the sol after hydrolysis. However, it was found that when the NH_3_•H_2_O is used as the basic catalyst, the gel time is stable, and the as-prepared SiBCO aerogel composites have stable properties.

## 4. Conclusions

Using TEOS as the silicon source, DEDMS as the carbon source, and TMB or PBA as the boron source, SiBCO gel precursors were synthesized through two steps of acid - alkali, and the SiBCO aerogel and its composites were prepared with mullite fibers as reinforcements. The structure and properties of the SiBCO aerogel and its composites with different boron sources or catalysts and ratios of raw materials were analyzed and characterized.

With the increase of the boron source amount, the content of boron in the SiBCO aerogel and its composites increased gradually, but the loss rate of boron also increased. The bending strength and compressive strength of SiBCO aerogel composites decrease with the increase of the TMB amount, but the strength can still meet the requirements of processing and usage. The increase of the TMB amount can improve the ability of SiBCO aerogel composites to maintain their thermal insulation at high temperature. When n (TMB)/n (TEOS) = 1, the composites have low thermal conductivity at high temperature.

The introduction of the boron element significantly improved the temperature resistance of SiBCO aerogel and its composites. With the increase of the boron source amount, the temperature resistance of the composites increased, and the service temperature of the composites could reach 1500 °C. The use of EN as the basic catalyst can better retain boron element in the SiBCO aerogel composites and improve its temperature resistance. When NH_3_•H_2_O is used as the basic catalyst, the gel time is more stable, and the synthesized composites have excellent properties. When n (NH_3_•H_2_O)/n (TEOS) = 0.09, the composites have the best temperature resistance.

## Figures and Tables

**Table 1:** Boron element content of SiBCO aerogel prepared with different basic catalysts.

	n(NH_3_·H_2_O)/n(TEOS)			n(EN)/n(TEOS)		
	0.06	0.07	0.08	0.06	0.07	0.08
Boron Element Content	1.78	1.64	1.61	2.29	2.16	2.09

**Table 2:** High-temperature thermal conductivity of SiBCO aerogel composites under vacuum.

Temperature/(°C)	1300	1400	1500
Thermal Conductivity/(W/(m·K))	0.1343	0.1382	0.1380
